# Bendable Polycrystalline and Magnetic CoFe_2_O_4_ Membranes by Chemical Methods

**DOI:** 10.1021/acsami.1c24450

**Published:** 2022-03-01

**Authors:** Pol Salles, Roger Guzmán, David Zanders, Alberto Quintana, Ignasi Fina, Florencio Sánchez, Wu Zhou, Anjana Devi, Mariona Coll

**Affiliations:** † ICMAB-CSIC, Campus UAB, Bellaterra, Barcelona 08193, Spain; ‡ School of Physical Sciences and CAS Key Laboratory of Vacuum Physics, University of Chinese Academy of Sciences, Beijing 100049, China; § Inorganic Materials Chemistry, Ruhr University Bochum, Universitätsstrasse 150, Bochum 44801, Germany

**Keywords:** CoFe_2_O_4_, Sr_3_Al_2_O_6_, sacrificial layer, atomic layer deposition, solution processing, flexible device

## Abstract

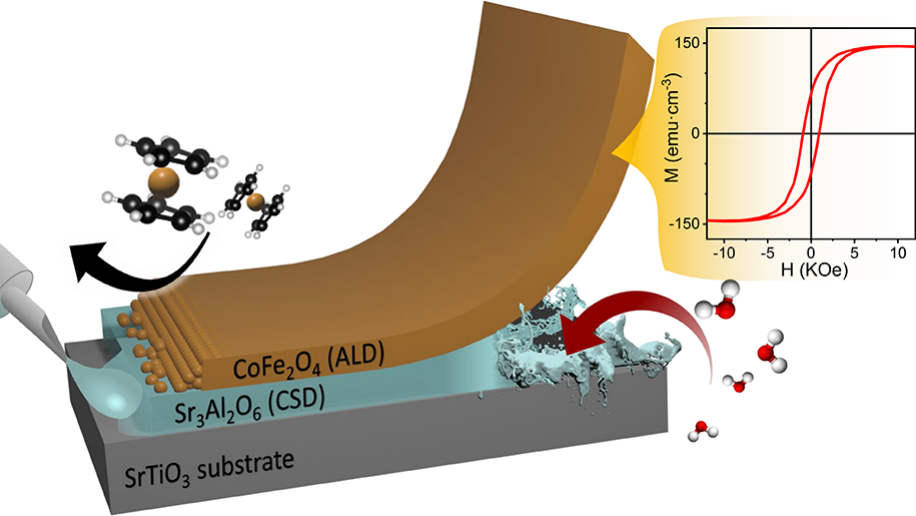

The preparation and manipulation of crystalline yet bendable functional complex oxide membranes has been a long-standing issue for a myriad of applications, in particular, for flexible electronics. Here, we investigate the viability to prepare magnetic and crystalline CoFe_2_O_4_ (CFO) membranes by means of the Sr_3_Al_2_O_6_ (SAO) sacrificial layer approach using chemical deposition techniques. Meticulous chemical and structural study of the SAO surface and SAO/CFO interface properties have allowed us to identify the formation of an amorphous SAO capping layer and carbonates upon air exposure, which dictate the crystalline quality of the subsequent CFO film growth. Vacuum annealing at 800 °C of SAO films promotes the elimination of the surface carbonates and the reconstruction of the SAO surface crystallinity. Ex-situ atomic layer deposition of CFO films at 250 °C on air-exposed SAO offers the opportunity to avoid high-temperature growth while achieving polycrystalline CFO films that can be successfully transferred to a polymer support preserving the magnetic properties under bending. Float on and transfer provides an alternative route to prepare freestanding and wrinkle-free CFO membrane films. The advances and challenges presented in this work are expected to help increase the capabilities to grow different oxide compositions and heterostructures of freestanding films and their range of functional properties.

## Introduction

The rapid development of electronic devices, telecommunication systems, and sensors pushes new functional demands with increasingly stringent requirements like flexibility, light weight, and miniaturization.^1,2^ Transition metal oxides present the richest variety of functional properties due to the large diversity of chemical compositions and structures that they can offer.^3−5^ An important twist is the processing of such materials as high-temperature growth conditions and specific crystalline substrates are required to achieve a certain degree of crystallinity, limiting their application field. The scientific community is putting a huge effort on learning how to grow these films and heterostructures to meet the new requirements while keeping their functionality.^5,6^ Mechanical exfoliation^7−9^ and wet and dry etching release methods^10^ are the most common processes for fabricating pliable and freestanding functional membranes. One of the most attractive approaches is the use of a sacrificial layer which allows one to detach the functional complex oxide film from the substrate. The choice of the sacrificial layer is of paramount importance. Its crystal structure, chemical composition, surface morphology, and lattice parameter will affect the crystalline quality of the transferred film and the selective etching.^11−14^ (La,Sr)MnO_3_ (LSMO),^15^ SrRuO_3_ (SRO),^16^ and Sr_3_Al_2_O_6_ (SAO)^17^ are some of the sacrificial layers used to prepare epitaxial perovskite oxide membranes such as SrTiO_3_,^18^ BiFeO_3_,^19,20^ BaTiO_3_,^21^ BaSnO_3_,^12^ SRO,^22,23^ and LSMO^17^ or even nanocomposites BaTiO_3_–CoFe_2_O_4_.^24^ Among the above-mentioned sacrificial layers, SAO is especially suitable to prepare perovskite oxides, it can be easily dissolved in water, contributing to the sustainability of the process, and by scrupulous variation of its lattice constant via cation substitution permits easy lattice matching with the functional oxide and avoid further crack formation.^12,14,25^ Nonetheless, the soft and open structure of SAO has a strong sensitivity to air humidity and can also facilitate cation interdiffusion during the high-temperature growth of the targeted complex oxide film.^11,17,26−28^ These characteristics can jeopardize the quality of the oxide membrane. Finally, the use of SAO to fabricate membrane oxides with dissimilar crystal structure has remained barely explored.

Generally, reported complex oxide membranes are prepared at high temperature by high-vacuum deposition techniques,^11,29^ although many challenges still remain in the continuous search for an ubiquitous and green route to prepare them. Today, it is possible to grow a wide variety of complex oxide thin films by cost-effective low-vacuum deposition methods^30−33^ offering a great opportunity to go one step further and investigate the viability of such techniques to obtain complex oxide membranes.

CoFe_2_O_4_ (CFO) has stimulated considerable interest for its remarkable magnetic and electrochemical properties.^24,34−38^ The possibility to provide mechanical flexibility to this material could dramatically increase its application area, for example, in wearable products,^39^ bendable magnetic sensors for diagnostics and medicine,^5,40−42^ and energy-related applications.^43^ The preparation of CFO films on pliable substrates and as freestanding membranes by high-vacuum deposition techniques has been attempted by direct growth on muscovite substrates,^44,45^ mechanical lift off,^7^ and in-situ growth on the rock salt MgO sacrificial layer.^46^ However, sustainable strategies to prepare a CFO freestanding membrane using water-soluble sacrificial layers and subsequent ex-situ and low-temperature growth to be further integrated in arbitrary substrates can open new areas of research.

In this exciting scenario, here we explore the ex-situ chemical synthesis of crystalline inverse spinel CFO bendable membranes using water-soluble SAO as a sacrificial layer. To reduce the typical high-temperature growth of these functional oxides and mitigate the cation interdiffusion at the CFO/SAO interface, low-temperature atomic layer deposition (ALD) is combined with solution processing. The critical effect of SAO air exposure on its surface structure and chemical composition has been assessed by reflection high-energy electron diffraction (RHEED), X-ray photoelectron spectroscopy (XPS), and scanning transmission electron microscopy (STEM). In-situ vacuum annealing of SAO films has been proved successful to improve its surface quality. Finally, we studied the exfoliation of the ex-situ grown ALD-CFO films and investigated the magnetic properties of the resulting bendable polycrystalline CFO membranes by superconducting quantum interference device (SQUID) magnetometry.

## Experimental Section

**Figure 1:**
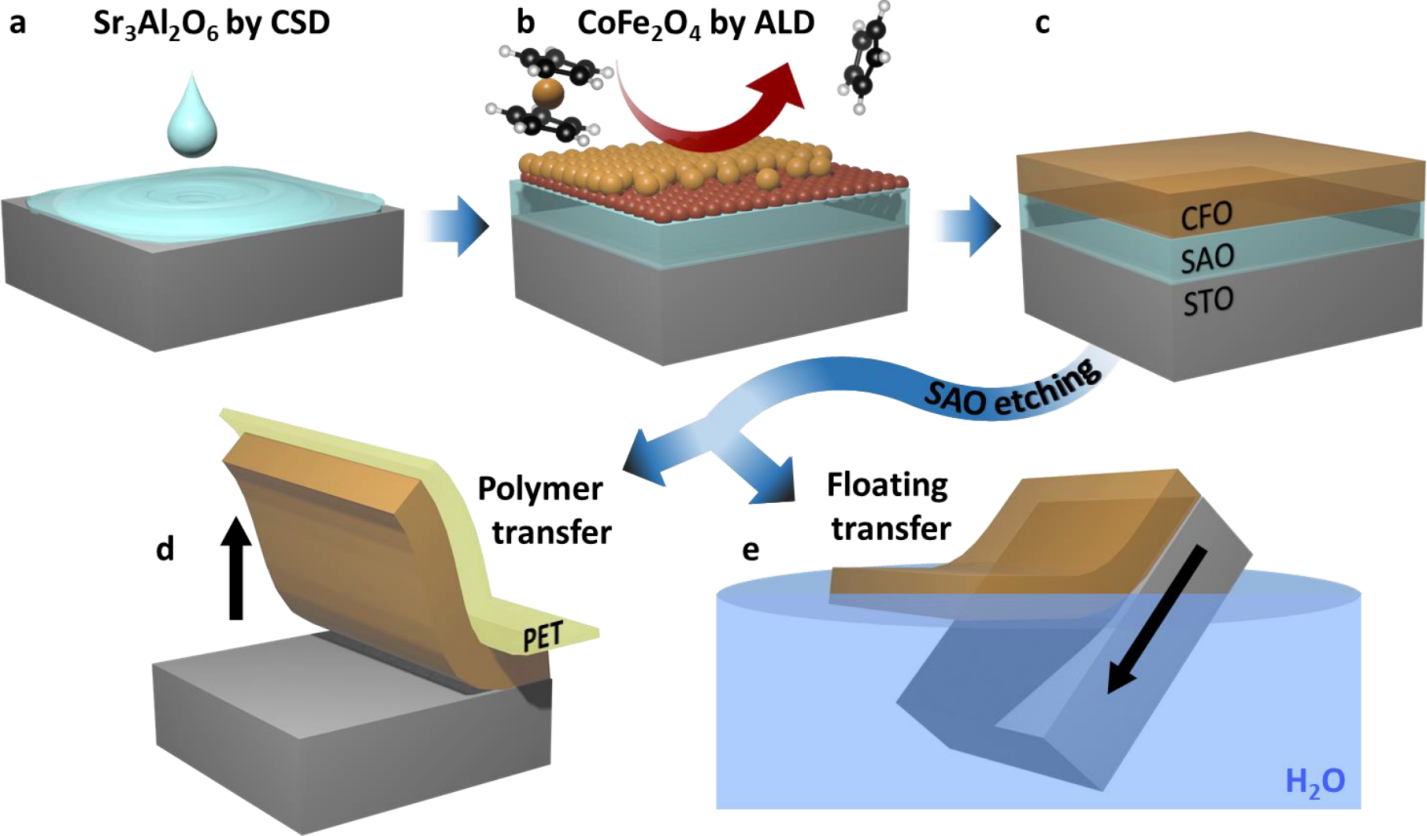
Sketch of the process followed to achieve CFO membranes. (a) SAO is deposited by chemical solution deposition on a (001) STO substrate. (b) CFO is deposited by ALD, achieving (c) a CFO/SAO//STO heterostructure. This heterostructure is immersed in water to finally transfer a CFO membrane following (d) the polymer support strategy and (e) the floating strategy.

The synthesis of the CFO membranes has been pursued following the sketch in Figure 1. The first step consists of the preparation of a SAO sacrificial layer on SrTiO_3_ (STO) by chemical solution deposition (CSD), followed by the preparation of the CFO on SAO//STO by ALD (lattice mismatch 7%, Figure S1), and finally perform the selective etching and subsequent exfoliation by means of either the use of a polymer support or the floating approach, as described in detail below. ALD has been chosen over CSD to prepare the CFO films because the deposition of a metal–organic precursor solution on SAO contributes to its degradation according to the above-mentioned SAO characteristics and permits low-temperature growth (250 °C).^47^

### Synthesis of SAO Sacrificial Layer

The SAO epitaxial layer was prepared by CSD on (001) STO substrates using metal nitrate solutions of 0.1–0.25 M as described elsewhere.^33^ Upon thermal treatment in a tubular furnace at 800 °C for 30 min with a heating/cooling rate of 25 °C·min^–1^ and under 0.6 L·min^–1^ O_2_ flow, the samples were sealed under vacuum to minimize surface degradation.

### Synthesis of CFO Films

CFO thin films were prepared by ALD with a Savannah 100 ALD system from Cambridge NanoTech Inc. Prior to film growth, the samples were exposed to ozone pulses to activate the surface forming a hydroxyl-terminated surface and facilitate the chemical reaction with the precursors. The deposition chamber was kept at 250 °C under a continuous N_2_ flow of 50 sccm. The metal–organic precursors were handled under inert atmosphere. [Co(Cp)_2_], bis(cyclopentadienyl)cobalt(II), was used as purchased and heated at 90 °C. [Fe(^*i*^pki)_2_], bis(*N*-isopropyl ketoiminate)iron(II), was synthesized as reported^48^ and heated at 130 °C. The tailor-made [Fe(^*i*^pki)_2_] was chosen over other commercial Fe precursors because of the good reproducibility and deposition control identified in previous works.^49^ The pulse of the metal–organic precursors was done in a pressure-boost mode as explained elsewhere.^49^ Ozone, O_3_, was used as the oxygen source with a pulse/purge duration of 0.2 s/10 s. To achieve stoichiometric Co:Fe = 0.5 corresponding to CoFe_2_O_4_, [Co(Cp)_2_] and [Fe(^*i*^pki)_2_] were pulsed in a supercycle approach with 5 subcycles of Co–O for 13 subcycles of Fe–O; [[Co(Cp)_2_]–O_3_ × 5 + [Fe(^*i*^pki)_2_]–O_3_ × 13)] × supercycle. A growth incubation period was identified for films thinner than 40 nm. Films thicker than 40 nm show a constant growth rate of 0.8 nm·(supercycle)^−1^ similar to what has been previously reported for ALD-CFO.^47,50,51^ A postannealing treatment was performed on some of the CFO thin films in a tubular furnace at 350–750 °C for 1 h under 0.6 L·min^–1^ O_2_ flow, as indicated in the text.

### Transfer of CFO Membranes

The transfer of the CFO thin films deposited on SAO//STO was done following two different strategies, polymer support and float on,^21,52^ as shown in Figure 1d and 1e, respectively. In this work, the polymer support strategy consists of adhering a polyethylene terephthalate (PET) polymer film to the CFO/SAO//STO heterostructure by applying slight pressure before immersing it in Milli-Q water. Once the SAO is etched in water, polymer and STO are mechanically separated, achieving a CFO membrane on the polymer support. For specific analysis, the CFO membrane held on PET has been subsequently transferred to a second support including Kapton and silicon.

On the other hand, the floating strategy is based on direct immersion of the CFO/SAO//STO heterostructure in water with no support. Once the SAO is etched, the sample is removed, left to air dry, and slowly immersed again in water, which helps in separating the CFO from the STO substrate by capillary forces, achieving a CFO freestanding membrane floating on the water surface. The floating CFO membrane can be directly fished with an arbitrary support (see Video S1 in the Supporting Information).

### Characterization

#### Crystal Structure

X-ray diffraction (XRD) θ–2θ and grazing incidence XRD (GIXRD) measurements were performed using a Siemens D-5000 and a Bruker-AXS (model A25 D8 Discover), respectively, both equipped with a Cu anode (Cu Kα = 1.5418 Å). The first one is adopted to study the epitaxial growth of the films and the second one to perform surface-specific phase analysis. Aberration-corrected scanning transmission electron microscopy (STEM) imaging was performed using a Nion HERMES-100, operated at 60 kV, at the University of Chinese Academy of Sciences, Beijing, China. High-angle annular dark-field (HAADF) images were acquired using an annular detector with a collection semiangle of 75–210 mrad. Cross-sectional STEM specimens were prepared using the standard focused ion beam (FIB) lift-out process in a Thermo Fisher Scientific FIB system. Protective amorphous carbon and thin Pt layers were applied over the region of interest before milling. To minimize the sidewall damage and sufficiently thin the specimen for electron transparency, final milling was carried out at a voltage of 2 kV. To reduce possible beam-induced structural damage on the SAO films, images were acquired with a reduced beam current (10 pA) and pixel dwell time (2 μs·px^–1^).

High-pressure reflection high-energy electron diffraction (RHEED) was performed with incidence of electrons along the [100] STO at a glancing angle of 1–2°. To study the crystalline evolution of SAO with temperature, RHEED patterns were acquired from as-deposited and in-situ-annealed films up to 825 °C at an oxygen partial pressure PO_2_ of 0.1 mbar for 30 min.

#### Film Thickness

The CFO film thicknesses were extracted from X-ray reflectometry (XRR) measurements using a Siemens diffractometer D-5000, and it was further validated with spectroscopic ellipsometry measurements using a GES5E Ellipsometer from SOPRA Optical Platform. In both cases, the CFO//STO samples were used as the reference.

#### Surface Morphology

Magnified optical images of the thin films and the corresponding membranes were taken by a Leica DM1750 M optical microscope. The surface morphology and roughness were studied by topographic images acquired by a Keysight 5100 atomic force microscopy (AFM) instrument and analyzed by Mountains8 software. Surface analysis and qualitative chemical composition were further investigated by scanning electron microscopy (SEM) using a SEM QUANTA FEI 200 FEG-ESEM equipped with energy-dispersive x-ray spectroscopy (EDX).

#### Surface Chemical Composition

X-ray photoelectron spectroscopy (XPS) measurements were performed with a SPECS PHOIBOS 150 hemispherical analyzer (SPECS GmbH, Berlin, Germany) using a monochromatic Al Kα radiation (1486.74 eV) source at 300 W at the Institut Català de Nanociència i Nanotecnologia (ICN2), Barcelona, Spain. The samples were analyzed with a spot size of 3.5 mm × 0.5 mm at a base pressure of 4 × 10^–10^ mbar. Pass energies of 20 and 50 eV and step sizes of 0.05 and 1 eV were used for the high-resolution and survey spectra, respectively. In-situ annealing to study the surface chemical composition with temperature (room temperature up to 800 °C) was performed in an annexed XPS chamber. The acquired spectra were processed with CasaXPS software using Shirley background subtraction. Binding energies were calibrated using Al 2p.

#### Magnetic Properties

The magnetic properties of the CFO thin films and membranes were probed employing a MPMS3 SQUID magnetometer from Quantum Design. In-plane magnetic hysteresis loops, *M(H)*, were acquired at 300 K with a maximum applied field of 15 kOe. To study the effect of the bending on the magnetic behavior of the CFO membranes, these were transferred to Kapton and clamped on plastic holders with different outward bending radii (*r*), flat, 5 mm, and 2.5 mm. To calculate the strain (ε) generated from the outward bending of the CFO membranes on kapton, it was used the equation ε = (*t*_CFO_ + *t*_Kapton_)/2*r*,^45,46^ where *t* is the thickness and *r* the curvature radius.

## Results and Discussion

### Chemically Deposited Oxide Heterostructure

**Figure 2:**
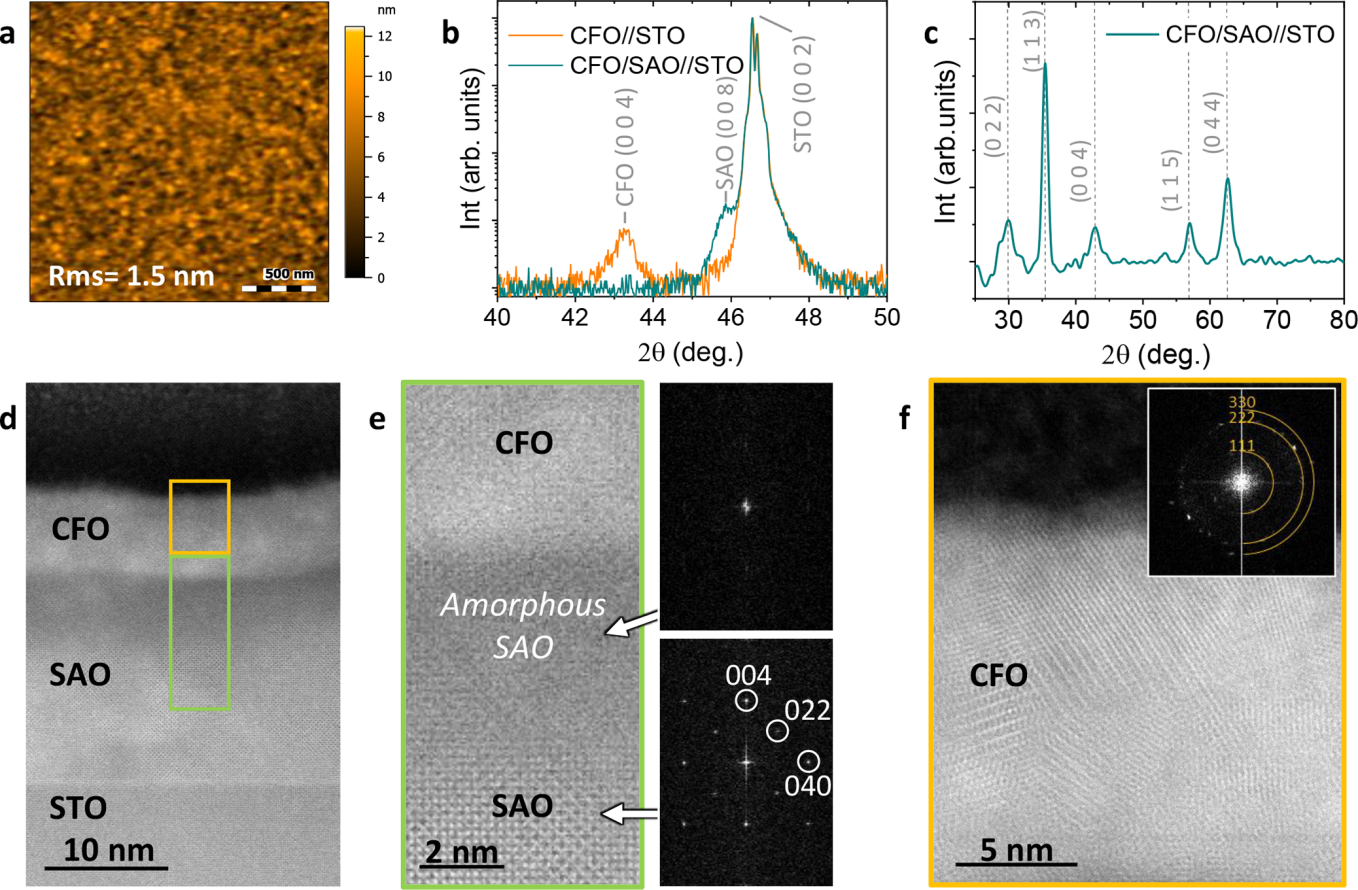
CFO/SAO//STO structure and surface morphology. (a) AFM topographic image of the CFO film. (b) XRD θ–2θ scan of the as-deposited CFO thin film (250 °C) on SAO//STO compared to CFO grown directly on STO substrate, (004) CFO, (008) SAO, and (002) STO Bragg reflections are indicated. (c) GIXRD of the as-deposited CFO/SAO//STO heterostructure. Indexed peaks correspond to the CoFe_2_O_4_ crystalline phase. (d) *Z*-contrast HAADF-STEM cross-section of the CFO/SAO//STO films. (e) Magnification of the interface area with the corresponding FFT from the amorphous part and the crystalline part. (f) Magnification of the CFO film with its corresponding FFT pattern.

The ALD-deposited 40 nm CFO on SAO//STO at 250 °C shows a homogeneous and smooth surface in Figure 2a, replicating the same morphology of SAO films (Figure S2) as expected from the conformal nature of the ALD technique. The structure and crystalline quality of CFO on SAO//STO have been studied by XRD and compared to the model system CFO//STO (Figure 2b). From the XRD pattern it can be identified that the CFO//STO sample presents two main Bragg reflections at 43.2° and 46.5° which correspond to (004) CFO and (002) STO, respectively, revealing that *c*-axis-oriented CFO films are obtained on single-crystal STO (001) substrates. On the other hand, for the CFO/SAO//STO sample, Bragg reflections centered at 45.8° and 46.5° correspond to (008) SAO and (002) STO, respectively, confirming the *c*-axis-oriented growth of SAO on STO, in agreement with previous work.^33^ However, the absence of the (004) CFO Bragg reflection in the latter indicates that no preferred (001) oriented growth is achieved in CFO. GIXRD analysis, which strengthens the signal from the first few nanometers of the film over the bulk/substrate, was done on CFO/SAO//STO films, see Figure 2b, disclosing that the CFO is crystalline and randomly oriented on SAO. The films do not present extra secondary phases as shown in Figure S3.

*Z*-contrast STEM imaging of a 10 nm ALD-CFO film on SAO//STO was selected to further study the heterostructure and interface quality with atomic resolution. From the low-magnification *Z*-contrast image (Figure 2d) it is observed that the ALD-CFO film is homogeneous and conformal on the SAO film. A closer look at the CFO/SAO interface (Figure 2e) identifies two different regions in the SAO film. The upper part in contact with CFO shows an amorphous SAO “capping layer”, while the bulk of the film is highly crystalline, further confirmed by the respective fast Fourier transform (FFT) patterns. Turning to the CFO film, higher magnification *Z*-contrast imaging shows the presence of randomly oriented crystalline CFO grains (Figure 2f) in agreement with the GIXRD analysis shown above. Attempts to restore the SAO surface quality in as-prepared films by acid etching or by extended O_3_ exposure at 250 °C in vacuum (10^–2^ Torr) before the ALD-CFO deposition were not successful.

**Figure 3:**
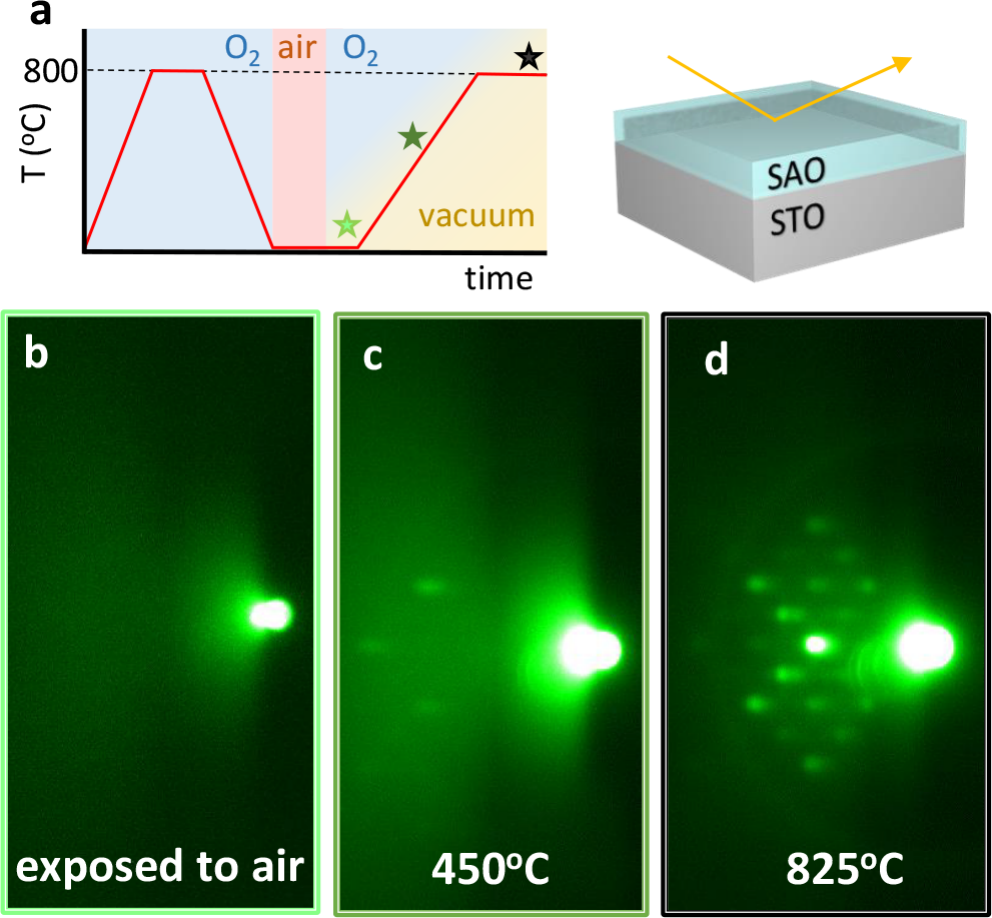
RHEED analysis of the SAO surface after air exposure. (a) Schematic of the followed thermal profile: SAO film is initially annealed in a tubular furnace at 800 °C with O_2_ flow to obtain epitaxial SAO; then it is cooled down, exposed to air, and annealed again in the RHEED chamber under vacuum with PO_2_. Colored stars in the temperature profile in (a) identify the temperature the measurements were performed: (b) at room temperature, (c) at 450 °C, and (d) at 825 °C.

In order to shed light on the formation and composition of this amorphous layer, RHEED and XPS analyses were carried out on bare as-deposited SAO films upon air exposure and after in-situ annealing in vacuum up to 825 °C, see sketch of the sample and thermal profile in Figure 3. Further details are provided in the Experimental Section. Figure 3b–d shows the high-pressure RHEED patterns from the as-deposited SAO film after air exposure and upon in-situ annealing taken along the [100] STO direction. From the pattern acquired from the as-deposited sample exposed to air, no diffraction spots are identified, revealing that the very first layers of the film are amorphous. Upon in-situ heating the sample at 450 °C in O_2_, low-intensity spots appear, indicating some degree of crystallization. Further in-situ annealing to 825 °C shows a spotty diffraction pattern corresponding to a crystalline SAO surface. Therefore, in-situ annealing in O_2_ causes the recrystallization of the SAO film surface.

**Figure 4:**
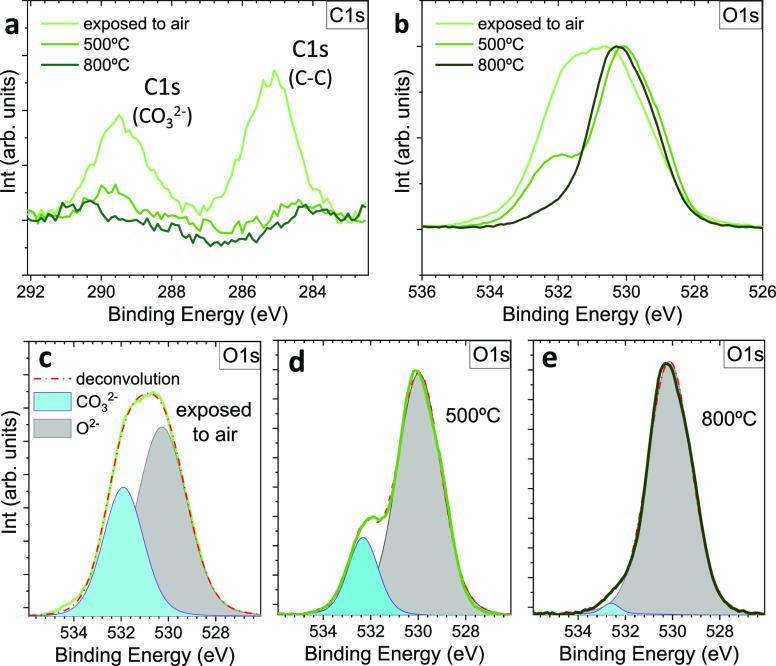
XPS analysis from a bare SAO surface. (a) C 1s and (b) O 1s core level spectra after air exposure and upon in-situ annealing in vacuum at 500 and 800 °C. O 1s spectra have been unbundled to clearly identify the different contributions (c) after air exposure, (d) at 500 °C, and (e) at 800 °C. Cyan area denotes carbonate species and gray area denotes lattice oxygen species.

In parallel, analogous XPS analyses were performed to elucidate the chemical composition of this amorphous top surface and its in-situ evolution when heated in vacuum (no O_2_ present in this case) following the thermal profile depicted in Figure 3a. From the overview spectrum (Figure S4) Sr, Al, C, and O can be identified. The Al 2p and Sr 3d core level spectra are not altered upon being exposed to the different in-situ annealing (not shown), from which the Sr:Al cation ratio can be easily calculated resulting in a stoichiometric ratio of 1.5. On the other hand, C 1s and O 1s core level spectra show significant differences, see Figure 4a and 4b, respectively. The C 1s core level spectrum from the as-deposited film shows two intense peaks, a broad one at 285 eV which extends up to 287 eV corresponding to the C–C with C–O–C contribution and another one at 289 eV assigned to the presence of the O–C=O moiety.^53,54^ When the sample is in-situ annealed in vacuum at 450 °C, the graphitic carbon vanishes and the C–O-related peak strongly decreases in intensity. A further in-situ annealing to 800 °C completely eliminates the C. The O 1s core level spectra in Figure 4b show a broad peak at 531 eV that gradually narrows upon annealing. Detailed analysis of the O 1s at each stage is shown in Figure 4c–e. The broad peak of the as-deposited sample centered at 531 eV can be deconvoluted into two main peaks at 530 and 532 eV (Figure 4c). The peak at 530 eV corresponds to metal oxide bond (529–530 eV), while the peak with lower intensity located in the range of 532–533 eV corresponds to carbonates.^55,56^ Upon heating, the O 1s carbonate contribution dramatically decreases with a small shift to higher energies being almost nonexistent at 800 °C, consistent with the C 1s trend. Therefore, according to this analysis, the SAO surface is covered with carbonates. It is very likely that they form within seconds when SAO samples are exposed to air before being sealed in vacuum for further manipulation. This surface reactivity is triggered by the affinity of the large Sr^2+^ ions for the hydration that could simultaneously hydrolyze few [Al_6_–O_18_]^18–^ rings.^11,17^ The formation of such carbonates together with the amorphous cap layer explain the growth of polycrystalline CFO films. Nonetheless, performing an annealing in vacuum at 800 °C removes the presence of carbonates and restores the SAO surface crystallinity. It is envisaged that in-situ deposition of complex oxides on this restored SAO surface could enable epitaxial growth.

**Figure 5:**
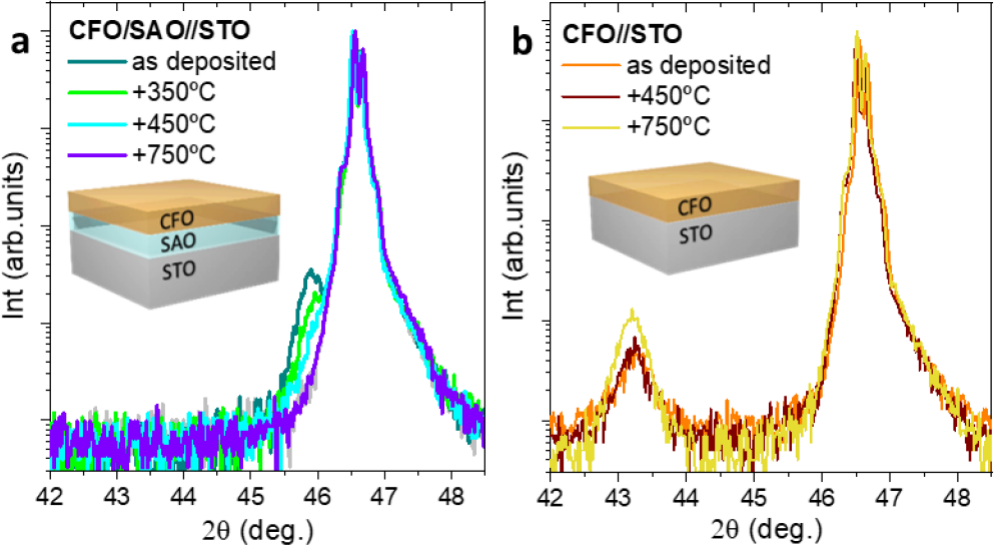
XRD θ–2θ analysis of as-deposited (250 °C) and ex-situ postannealed CFO films from (a) CFO/SAO//STO and (b) CFO//STO.

With the aim to study the effect of a postannealing temperature on the crystalline quality of CFO deposited on air-exposed SAO//STO, the heterostructure has been subjected to several ex-situ thermal treatments in a tubular furnaces from 350 to 750 °C under O_2_ flow (Figure 5a). It is observed that by increasing the postannealing temperature, the SAO (008) Bragg reflection decreases in intensity whereas no CFO (004) reflection appears. From GIXRD analysis, no significant changes are identified in the polycrystalline nature of CFO (Figure S5). An analogous study on the CFO//STO model system revealed that by increasing the postannealing temperature, the (004) CFO Bragg reflection increases in intensity, indicating that the crystalline quality does improve (Figure 5b).^51,57^ Therefore, it is very likely that the CFO/SAO interface plays a key role in the CFO/SAO//STO crystallinity. According to reported STEM analysis on the LSMO/SAO//STO system, which demonstrated the susceptibility of SAO for interface cation diffusion when exposed to high temperature,^26,27^ it is suggested that the observed decrease in intensity of the SAO (008) reflection could be due to interface cation diffusion between CFO and SAO. In addition, XPS studies performed on exfoliated ALD-CFO films revealed the presence of Sr and Al traces (Figure S6), which would reinforce the hypothesis of cation diffusion during the postannealing. Baek et al.^26^ overcame this issue by in-situ growing a few unit cells of the SrTiO_3_ buffer layer.

### CFO-Transferred Membranes

Upon Milli-Q water immersion of the CFO/SAO//STO heterostructure, two routes have been investigated to obtain CFO membranes: the use of a PET polymer support and the floating approach, as shown in Figure 1 d and 1e, respectively. Importantly, after CFO exfoliation, the STO substrate can be reused for subsequent experiments, contributing to the sustainability of the process.

#### Polymer Transfer

**Figure 6:**
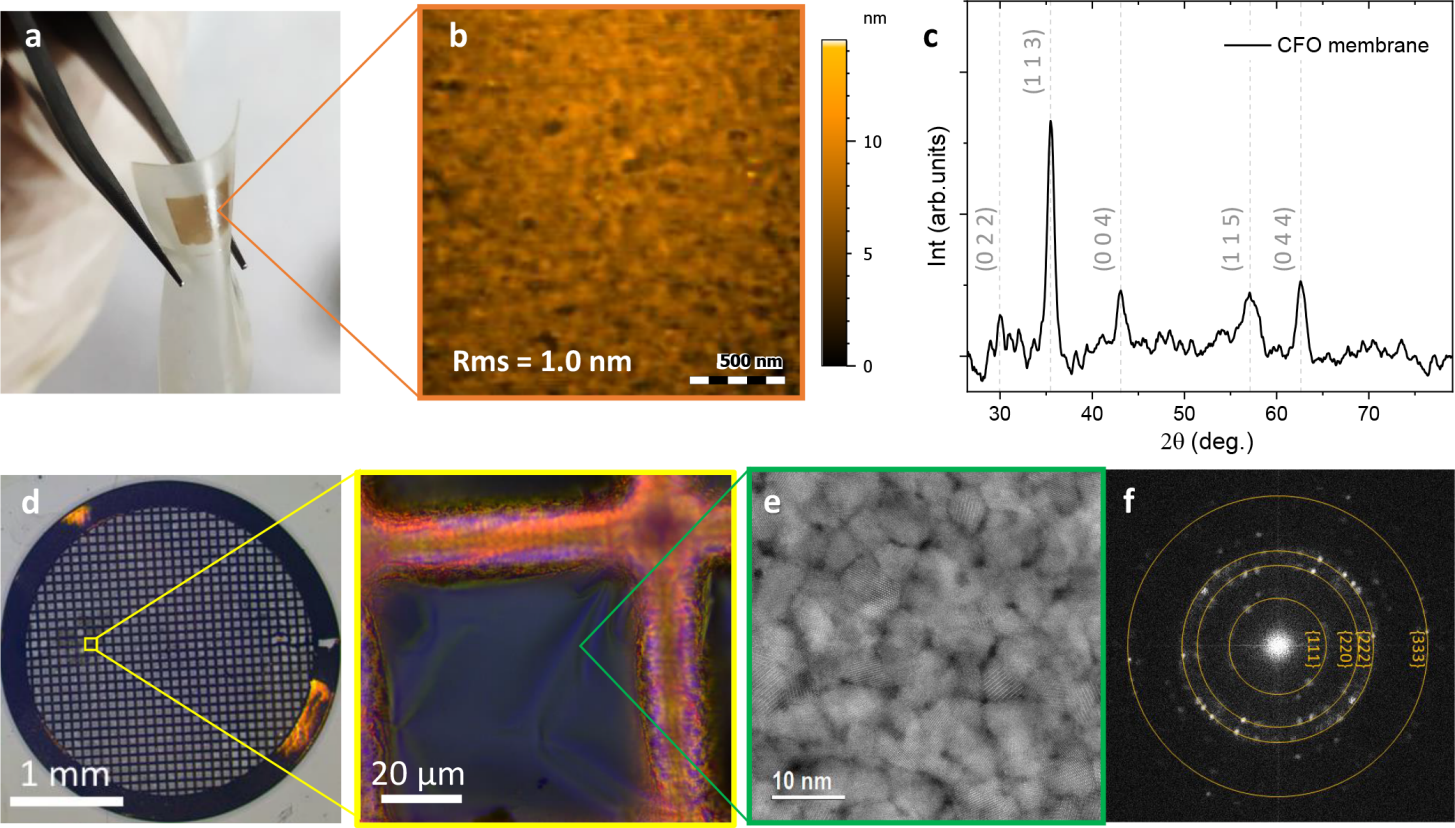
Structure and surface topographic characterization of 250 °C deposited CFO membranes and transferred by (a–c) a polymer support and (d–f) floating. (a) 5 × 5 mm^2^ CFO membrane on PET support with its corresponding (b) topographic analysis by AFM and (c) GIXRD analysis. (d) Optical microscope image of a Cu grid with a piece of CFO membrane picked directly from water with the corresponding magnification of the CFO membrane. (e) STEM top view of the CFO membrane and its corresponding (f) FFT pattern.

To achieve successful exfoliation of the entire CFO membrane, it is necessary to identify the optimal conditions to etch the sacrificial layer for the CFO/SAO//STO system. Note that the very thin film thickness of CFO makes the potential membrane susceptible to cracking and generating micro- and nanoscale defects during the exfoliation process.^52,58^ To improve the mechanical properties of the CFO membrane, attachment of a polymer support on the CFO prior to immersion of the heterostructure in water was first studied. Following this polymer strategy, CFO membranes of 5 × 5 mm^2^ have been successfully transferred to PET with no cracks, as shown in Figure 6a. The transferred CFO membranes remain strongly attached to the PET support by dispersive adhesion with good mechanical stability facilitating its manipulation and further characterization.^59^ The surface topography and structure of a 60 nm CFO membrane was characterized by AFM and GIXRD. Figure 6b shows the surface morphology studied by AFM topographic images of the CFO membrane on PET, which is proved to be smooth and homogeneous with a root-mean-square surface roughness (rms) of 1.0 ± 0.2 nm. GIXRD shows that the polycrystalline nature of the CFO film before exfoliation is preserved after the transfer (Figure 6c). We emphasize the feasibility of performing a second transfer of the CFO membranes from PET to another arbitrary substrate such as silicon wafers or kapton tapes, as shown in Figure S7.

#### Floating and Transfer

Another promising strategy that was studied to obtain freestanding CFO membranes is the floating transfer method, Figure 1e. In this case, the CFO/SAO//STO heterostructure is immersed in water with no additional support. Once the SAO has been completely etched, the remaining heterostructure is taken out to dry and then slowly immersed again in water with a given angle facilitating that the capillary forces of the water surface separate the CFO membrane from the STO substrate, leaving the CFO membrane floating; an example of the floating process is shown in Video S1. Finally, the CFO membrane can be picked out with a support. Figure 6d–f shows an example where a CFO membrane of 14 nm was picked out from the water surface with a Cu grid. The freestanding CFO membrane remains unbroken over the Cu grid holes as shown in Figure 6d, which allowed us to perform HR-STEM analysis. The crystalline grains of the CFO membrane can be distinguished with a diameter size ranging from 5 to 15 nm (Figure 6e). Different atomic planes of the crystalline CFO are identified, and the crystallinity is further confirmed by the FFT pattern presented in Figure 6f. A more extensive study of the crystallinity of the CFO grains observed by STEM can be found in Figure S8. This strategy presents some benefits when compared to the polymer transfer. First, the CFO membrane wrinkles formed during etching can easily spread when the membrane remains floating on water. As a result, flat and smooth membranes can be obtained when picked up with a support. Another advantage is that when the CFO floating membrane is picked out by a support, the adhesion of the membrane with the support is mainly related to the gravity and the drying of water between them, leaving the interface energy between the support and the CFO membrane irrelevant. This independence from the interfacial forces implies the freedom of using virtually any type of support without limitations, which is not the case for the polymer transfer strategy. However, on the other hand, a drawback of the floating transfer is that because the CFO membranes are so fragile and in this case no support is used during the etching and separation, cracks can be formed easily, compromising the integrity of the CFO membrane.

### Magnetic Properties

**Figure 7:**
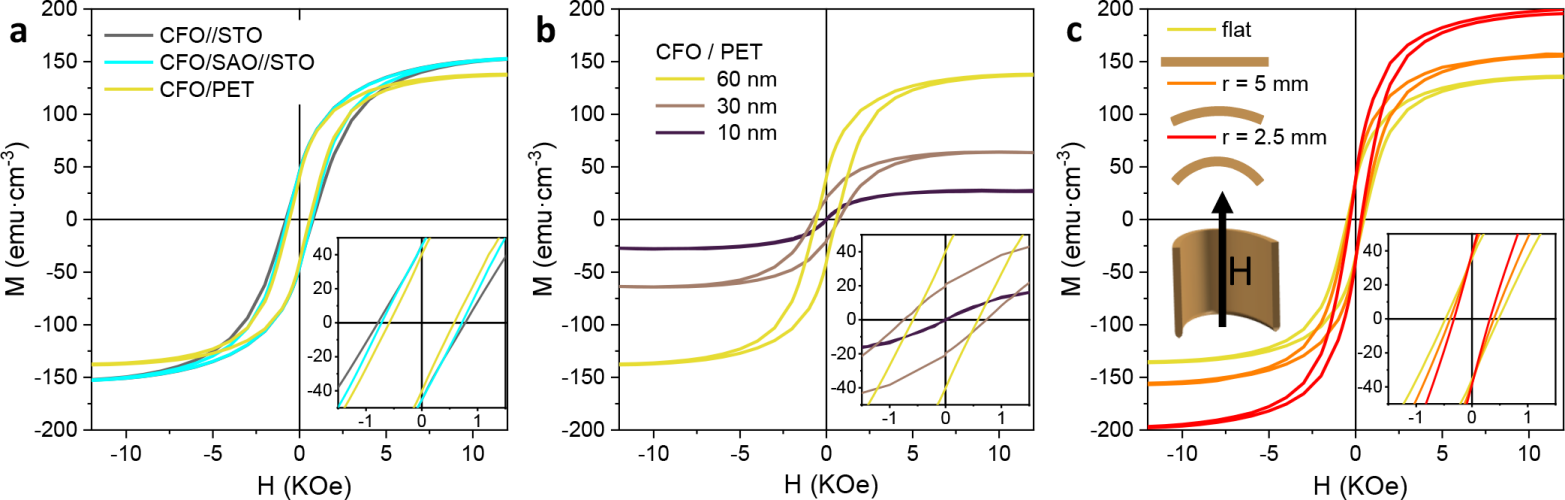
In-plane magnetic hysteresis loops, *M(H)*, performed at 300 K on CFO films and membranes. Inset shows *M(H)* from −1.5 to 1.5 kOe. (a) CFO films grown on STO, SAO//STO, and transferred onto PET. (b) CFO membranes on PET for different thicknesses, 10, 30, and 60 nm. (c) CFO membranes under different radii of curvature.

The magnetic properties of 60 nm CFO films and membranes at 300 K were assessed using SQUID magnetometry (Figure 7). In-plane *M(H)* hysteresis loops corresponding to CFO//STO, CFO/SAO//STO, and transferred CFO on PET are shown in Figure 7a. From these measurements it can clearly be seen that the inclusion of the SAO layer does not significantly alter the magnetic properties of CFO, showing a similar saturation magnetization, *M*_s_, of 150 emu·cm^–3^ and coercivities of 0.7 kOe. Moreover, after etching the SAO layer and CFO transfer to a PET substrate, the CFO/PET magnetic properties also remain unaltered as evidenced by the resemblance in the hysteresis shape and saturation magnetization. Note that the obtained magnetization values for the CFO membranes are congruous with earlier studies on epitaxial CFO membranes.^7,44−46^ These *M*_s_ values are lower than those for bulk CFO materials.^60^ Variations in *M*_s_ could arise from many different factors including off-stoichiometry, strain, grain boundaries, and structural distortions.^36,50,61^ Considering that in our work both epitaxial films and polycrystalline membranes show similar *M*_s_ values, this decrease could be tentatively attributed to cation migration and redistribution in the tetrahedral and octahedral sites, as previously demonstrated in spinel ferrite samples.^62−64^

Figure 7b shows the magnetic properties for transferred CFO membranes with thicknesses of 10, 30, and 60 nm onto PET substrates. The CFO thickness was tuned controlling the number of ALD cycles. The CFO membranes are homogeneous and continuous with no cracks. However, random wrinkles were observed (Figure S9). From the *M(H)* hysteresis loops in Figure 7b, it is observed that *M*_s_ decreases by diminishing the CFO membrane thickness. Notoriously, the 10 nm CFO membranes show a paramagnetic behavior. Cation diffusion and doping in a spinel structure could distort its structure, altering the magnetization of saturation.^65^ Therefore, it is very likely that the Al and Sr traces identified on the CFO membrane surface by XPS (Figure S6), probably due to the easy cation diffusion during the sample processing, could distort the CFO structure, diminishing *M*_s_. Thus, the thinner the membrane, the larger the contribution of Sr and Al and thus the lower the *M*_s_.

*M(H)* hysteresis loops from 60 nm CFO membranes transferred on kapton tape and held at different outward bending radii, *r* (flat, 5 mm, and 2.5 mm) were acquired. These bending radii correspond to 0, 0.75%, and 1.5% tensile strains, respectively, see the Experimental Section for further details. Figure 7c shows the magnetic field-dependent magnetization curves in the in-plane direction (parallel to the curvature axis) for the different bendings. The membranes show an increase in the *M*_s_ while *H*_c_ is barely modified by increasing the curvature radius, in agreement with previous reports on epitaxial CFO membranes.^46^ Note that despite the large amount of research performed on the magnetic behavior of epitaxial-strained rigid CFO films, it is only recently that the first reports appeared exploring the anisotropy–strain scenario on epitaxial pliable CFO membranes.^44−46^ Therefore, more detailed analysis is required to elucidate the mechanism by which the magnetization varies with bending in polycrystalline membranes.

## Conclusions

We investigated the synthesis of CFO membranes by combining atomic layer deposition and solution processing using a sacrificial layer of SAO. We shed light on the chemical preparation of oxide heterostructures under compatible thermodynamic conditions and the critical role of interface perfection on the crystalline quality of the CFO membranes. Surface-specific characterization on the structure (RHEED, STEM) and chemistry (XPS) of epitaxial SAO reveals the formation of an amorphous top layer and carbonates. In this study, we also unravel how to restore the SAO surface quality by annealing in vacuum. ALD-CFO growth at 250 °C on air-exposed solution-processed SAO is polycrystalline, and the CFO films can be easily transferred to a polymer support. Alternatively, CFO membranes have been prepared by floating, resulting in freestanding membranes. We demonstrated the formation of CFO membranes of various thicknesses (from 10 to 60 nm) with the possibility of being stretched or bent, showing robust magnetization at room temperature. This pliable system will allow future studies on the intrinsic effect of mechanical strain, which is of high interest for flexible magnetostrictive sensors or actuators but also for energy- and medicine-related applications. Therefore, this is an straightforward, sustainable, and cost-effective approach to prepare functional crystalline oxide membranes, opening the door for manufacturing a wide variety of oxide membranes and artificial architectures with less restricted and mild processing conditions. We also envisage new opportunities to produce oxide membranes with tuned degree of crystallinity when chemical methods are combined with high-vacuum deposition methods, broadening the study of physical and chemical phenomena occurring at these novel and bendable oxide interfaces for enhanced functionalities.

## Supporting Information Available

Atomic representations of the STO, SAO, and CFO crystalline structures; AFM analysis of STO, SAO//STO, and CFO//STO; XRD θ-2θ of CFO/SAO//STO; XPS overview of SAO//STO; GIXRD and AFM of CFO/SAO//STO after postannealing at 450 °C; XPS analysis of Sr and Al traces at CFO membranes; examples of second transfer of CFO membranes; XRR measurements of CFO//STO; optical microscopy images of CFO membranes of different thicknesses (PDF)
Video of the floating process of a CFO membrane (MP4)
