# Supplementary material for: Bendable Polycrystalline and Magnetic CoFe2O4 Membranes by Chemical Methods
Source: ACS Appl Mater Interfaces. 2022 Mar 1;14(10):12845–54. doi: 10.1021/acsami.1c24450 (PMC8931725; doi:10.1021/acsami.1c24450)
Supplement: Supplementary file 1 — am1c24450_si_001.pdf [file am1c24450_si_001.pdf]

Supporting Information for Bendable polycrystalline and magnetic $CoFe_2O_4$ membranes by chemical methods

Pol Salles,[†] Roger Guzmán,[‡] David Zanders,[¶] Alberto Quintana,[†] Ignasi Fina,[†] Florencio Sánchez,[†] Wu Zhou,[‡] Anjana Devi,[¶] and Mariona Coll[*,†]

†*ICMAB-CSIC, Campus UAB 08193, Bellaterra, Barcelona, Spain*

‡*School of Physical Sciences and CAS Key Laboratory of Vacuum Physics, University of Chinese Academy of Sciences, Beijing 100049, China*

¶*Inorganic Materials Chemistry, Ruhr University Bochum, Universitätsstraβe 150, Bochum 44801, Germany.*

E-mail: mcoll@icmab.es

Phone: +34 93 5801853

CFO on SAO//STO

Surface morphology and structure

$CoFe_2O_4$ (CFO) has a spinel ferrite structure AB_2O_4 (space group ***Fd3m***) with a lattice parameter of a=8.39 Å. SAO and STO are perovskites with a=15.84 Å and a=3.91 Å respectively. Therefore, epitaxial growth of CFO on these perovskites would imply a large-lattice mismatch of ∼ 6-7%, see **Figure S1**.

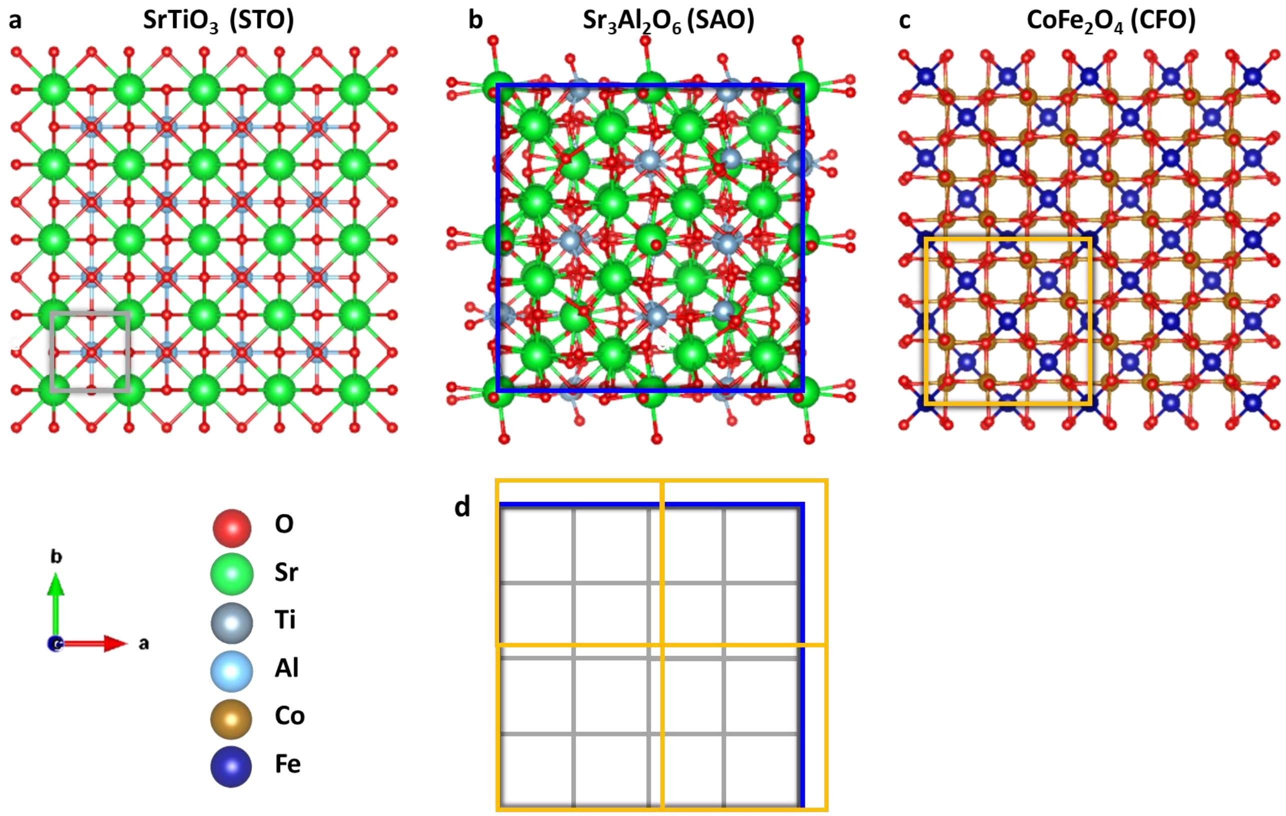

Figure S1: Atomic representation of (a) STO, (b) SAO and (c) CFO crystalline structures. (d) The cells are superposed to facilitate mismatch comparison.

The conformality of the CFO coatings made by Atomic Layer Deposition (ALD) can be confirmed with the surface morphology analyzed by Atomic Force Microscopy (AFM), see **Figure S2**. The monocrystalline (001) STO substrates are previously treated with HF and annealed at 950°C to achieve the terrace-like morphology (Figure S2a). [1,2] When the SAO sacrificial layer is deposited by chemical solution deposition (CSD) a smooth surface is

achieved, but the terrace-like morphology of the STO is lost (Figure S2b). On the other hand, depositing the CFO films by ALD, because of the atomic precision of the technique and the surface reaction-based mechanism, the coating is completely conformal keeping the surface topography of the previous layer, even the terrace-like morphology of the STO substrate (Figure S2c).

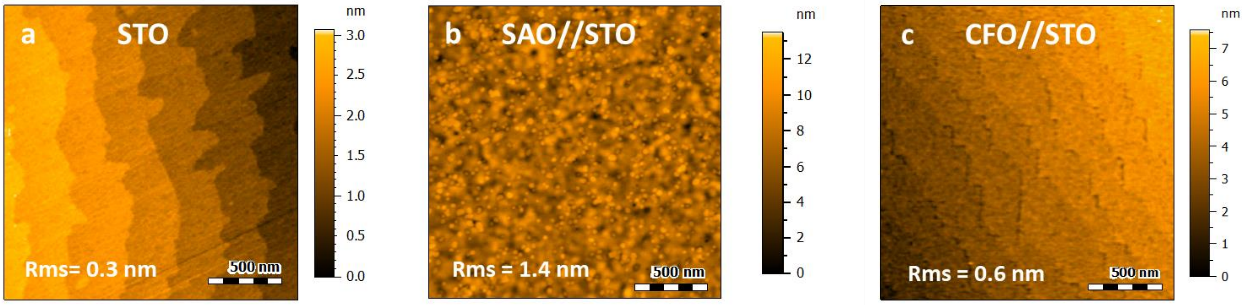

Figure S2: Surface morphology by AFM of (a) STO bare substrate, (b) SAO//STO and (c) CFO//STO.

The XRD analysis of the CFO film on SAO//STO and on bare STO show no secondary phases in the 2θ range of 20-80°, see **Figure S3**

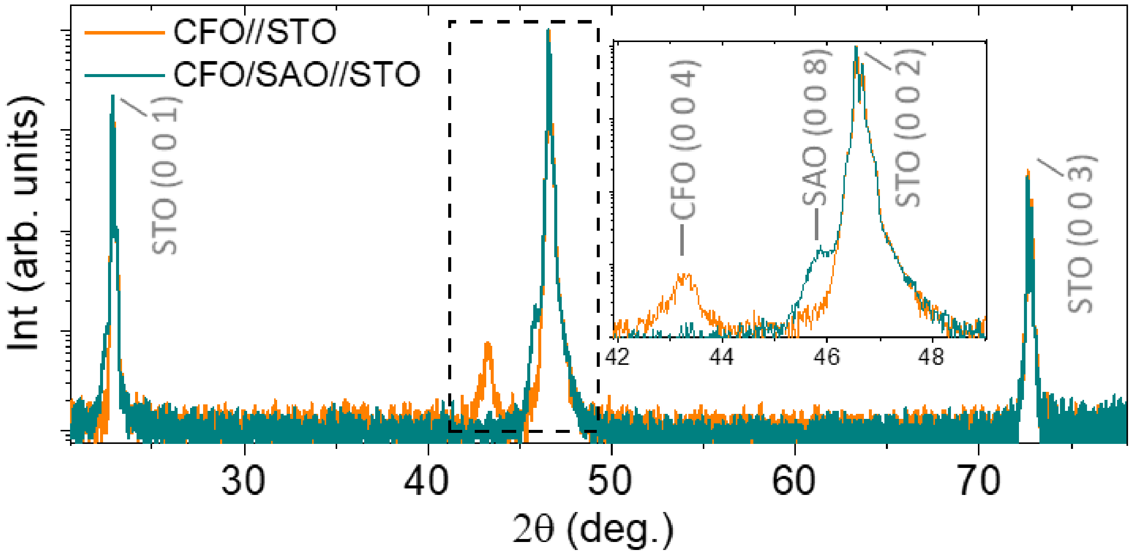

Figure S3: XRD θ -2θ scan of the as deposited CFO thin film on SAO//STO compared to CFO grown directly on STO substrate. Inset corresponding to the θ -2θ range of the (004) CFO, (008) SAO and (002) STO Bragg reflections.

Surface chemical composition

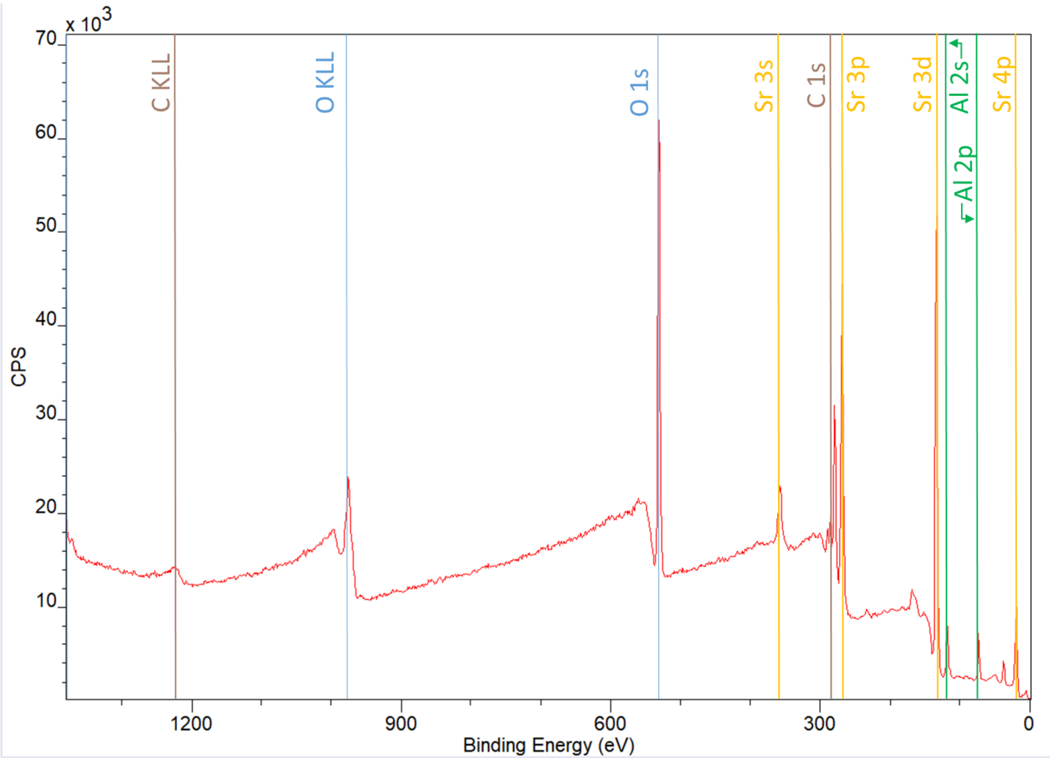

Figure S4: XPS overview analysis of SAO surface after air exposure

Post-annealing treatment on CFO/SAO//STO heterostructures

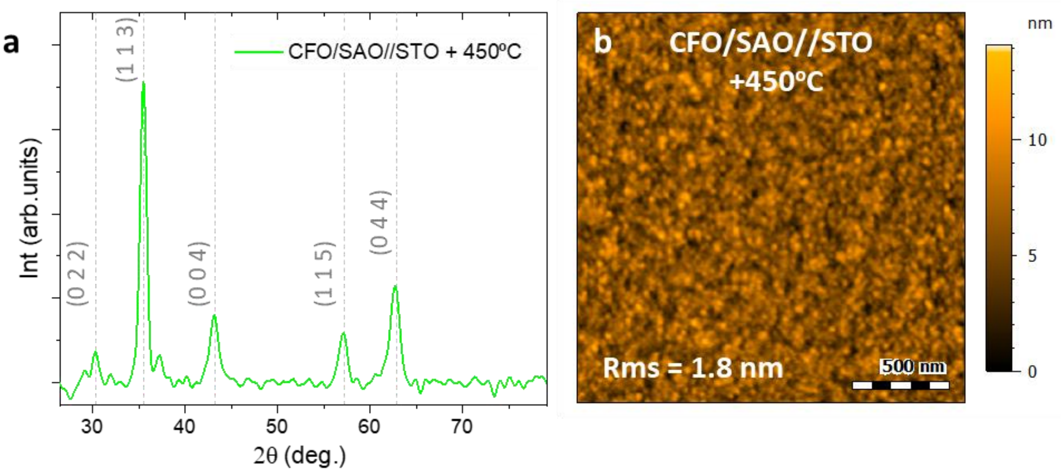

Figure S5: Effect of post-annealing treatment (1 h at 450°C) to CFO/SAO//STO heterostructures: (a) GIXRD 2θ scan, (b) AFM topographic image

CFO membranes

Surface chemical composition

As schematically shown in **Figure S6a**, the chemical composition of the CFO film at the CFO/SAO interface was studied after etching the SAO phase and transferring the CFO membrane to a PET support, thoroughly washed with DI water. In this way, the CFO membrane is turned upside down exposing the face previously in contact with SAO, which was characterized by X-Ray Photoelectron Spectroscopy (XPS) analysis. Note that XPS is a technique that allow to study the chemical composition of the very top surface of a material, with a depth profile lower than 10 nm. Therefore, analyzing the presence of Sr and Al would allow to determine if traces of SAO diffused into the first few nanometers of the CFO film. This characterization was done to two different CFO membranes to see the effect of the post-annealing treatment (as deposited *versus* 450°C post-annealing). Figure S6b shows the Sr3d spectra of the two samples, both with a peak at 134 eV confirming the presence of Sr in both of them, but with different intensities of the peak. The amount of Sr calculated in each case in percentage of the whole atomic contribution corresponds to 0.5 % for the as deposited and 1.5 % for the post-annealing. Figure S6c shows the Al2p spectra used to analyze the presence of Al in the CFO film. In this case both samples had similar peak intensities at 74 eV, corresponding to a 8 % of the atomic contribution. These results imply an accumulation of mainly Al at the CFO surface after SAO etching, which is independent of whether there was post-annealing or not. Sr, on the other side, tends to accumulate much less at the CFO film, and in this case it gets worsened by post-annealing the sample.

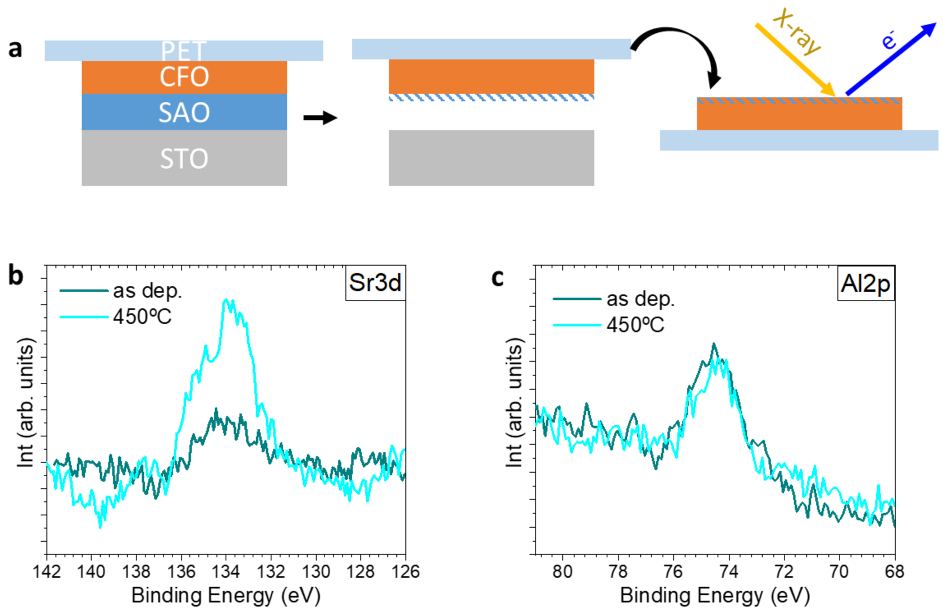

Figure S6: XPS analysis of the CFO surface after SAO etching. (a) Schematics of the process followed to study the CFO face that was initially in contact with SAO. (b) Sr3d core level spectra and (c) Al2p core level spectra of the CFO films as deposited and with a 450°C post-annealing in oxygen.

Double transfer CFO membranes

In the polymer support strategy, the transfer of CFO after SAO etching was done using a PET support. A PET support gives robustness and mechanical stability to the CFO membranes, while keeping flexibility and high optical transmittance to visible light.[3] However it presents some limitations when it comes to a few characterization techniques. For example, GIXRD measurements on PET are not possible because the background signal of the PET support hides the relatively weak reflections of the thin CFO membranes. SEM analysis of CFO//PET is also compromised because of the charging of the insulating PET support. Therefore, for some specific characterization, subsequent transfer to another substrate was required.

The adhesion between the CFO membrane and the PET is based on dispersive adhesion with no involvement of a chemical reaction, therefore this attachment is reversible and allows to easily transfer the CFO membrane to other supports.[3] **Figure S7** shows how CFO

membranes can be transferred twice, first to PET and then to another support, showing as examples a Si support with a SiO_2 layer on the top and a piece of Kapton tape. The smoothness of the CFO membrane (probed by AFM in Figure 6b, main manuscript), combined with the physical favorable interaction at the interface and some applied pressure, allow to successfully transfer most part of the CFO membrane to other supports. Schematics of the entire process from the initial CFO/SAO//STO heterostructure to the final CFO//Si or CFO//Kapton heterostructure are shown in Figure S7a. After transferring the CFO membrane to Si, it was characterized by SEM-EDX (Figure S7b,c). The membrane is dense and no cracks were identified in the area analyzed. From the EDX analysis, O, Si, Fe and Co were identified. Another example of a successful second transfer is Kapton tape. As shown in Figure S7d a 5 x 5 mm^2 CFO film can be entirely transferred from PET to Kapton.

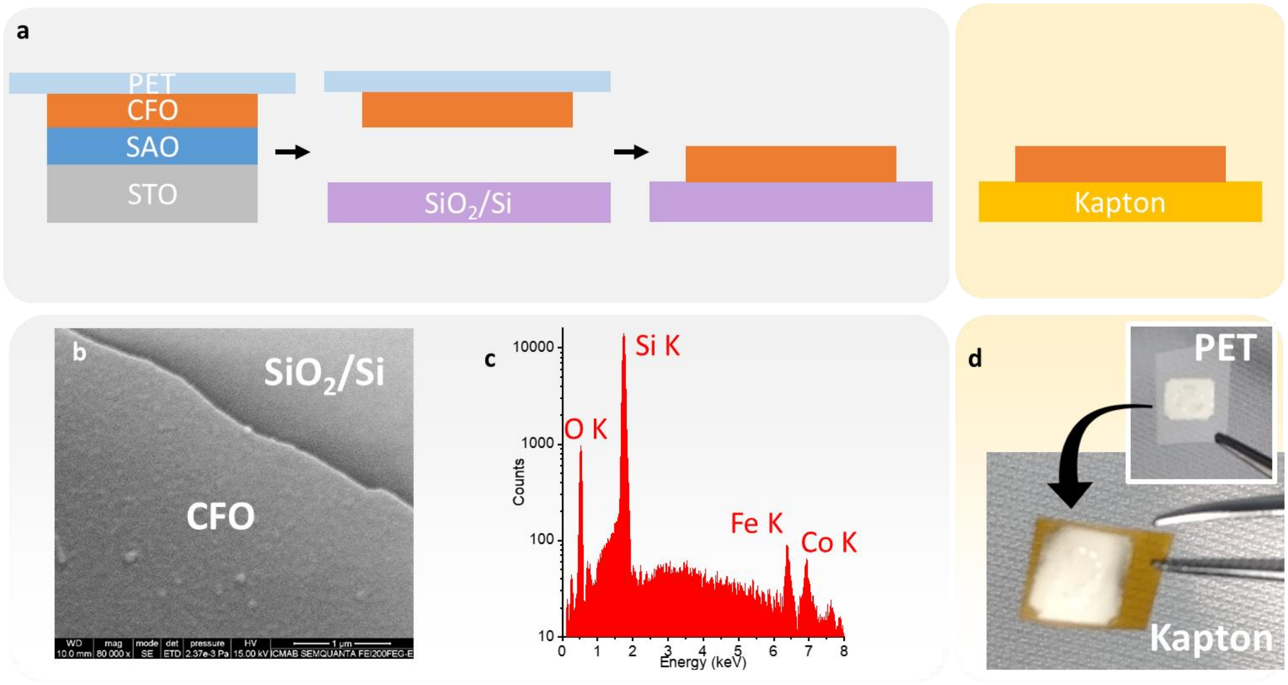

Figure S7: Second transfer of CFO membranes: (a) Scheme of the procedure to perform the second transfer of a CFO membrane from PET to either a SiO_2/Si or Kapton tape. The transferred CFO membrane to Si was analysed by (b) SEM and (c) EDX. (d) Picture of the CFO membrane entirely transferred to Kapton (5x5 mm^2).

Microstructure of freestanding CFO membranes

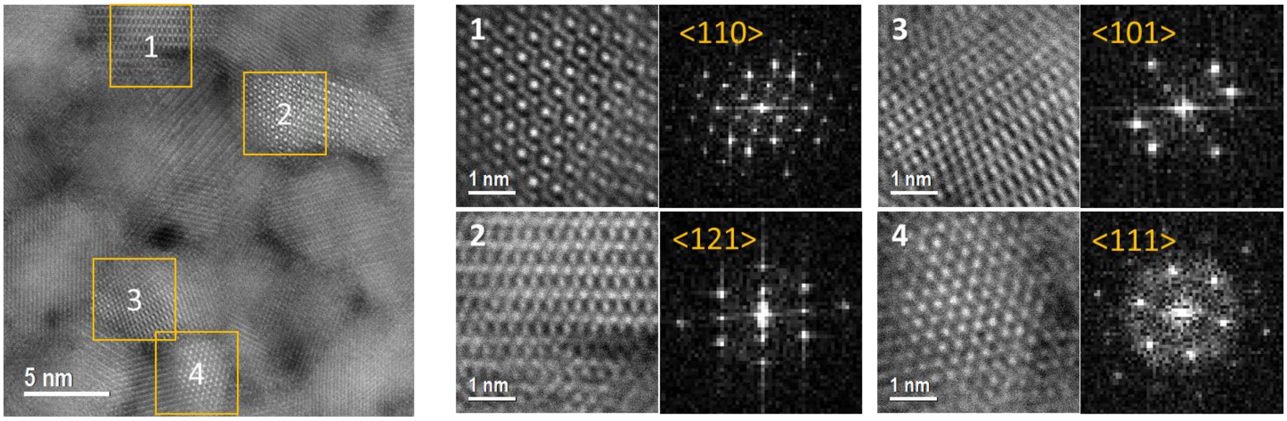

Figure S8: HR-TEM top-view analysis of a 14 nm freestanding CFO membrane. Identification of different crystallographic orientations of four grains with the corresponding fast fourier transform patterns.

CFO membranes with different thicknesses

Simultaneous ALD-CFO depositions on SAO//STO and on bare STO, allowed to use samples on STO as reference for thickness measurements. X-ray reflectivity (XRR) measurements were carried out on all the samples to confirm their thickness. **Figure S9**a shows the XRR spectra of the CFO//STO samples with 10, 30 and 60 nm thin, analogous to the CFO membranes analyzed in Figure 7 main manuscript. Kiessig fringes, used for thickness determination from XRR measurements, are well-defined and more easily analyzed for very low roughness thin films allowing to confirm the expected thickness according to the ALD cycles performed.

Figure S9b-d shows optical microscopy images of the transferred CFO membranes on PET for different CFO thicknesses. In all three cases, ripples along all the surface are observed. The size of these ripples appear to be directly related to the thickness of the membrane, as proportionally schematized under the optical microscope images. The appearance of these features could be related to a relaxation of the film after substrate declamping.[4,5]

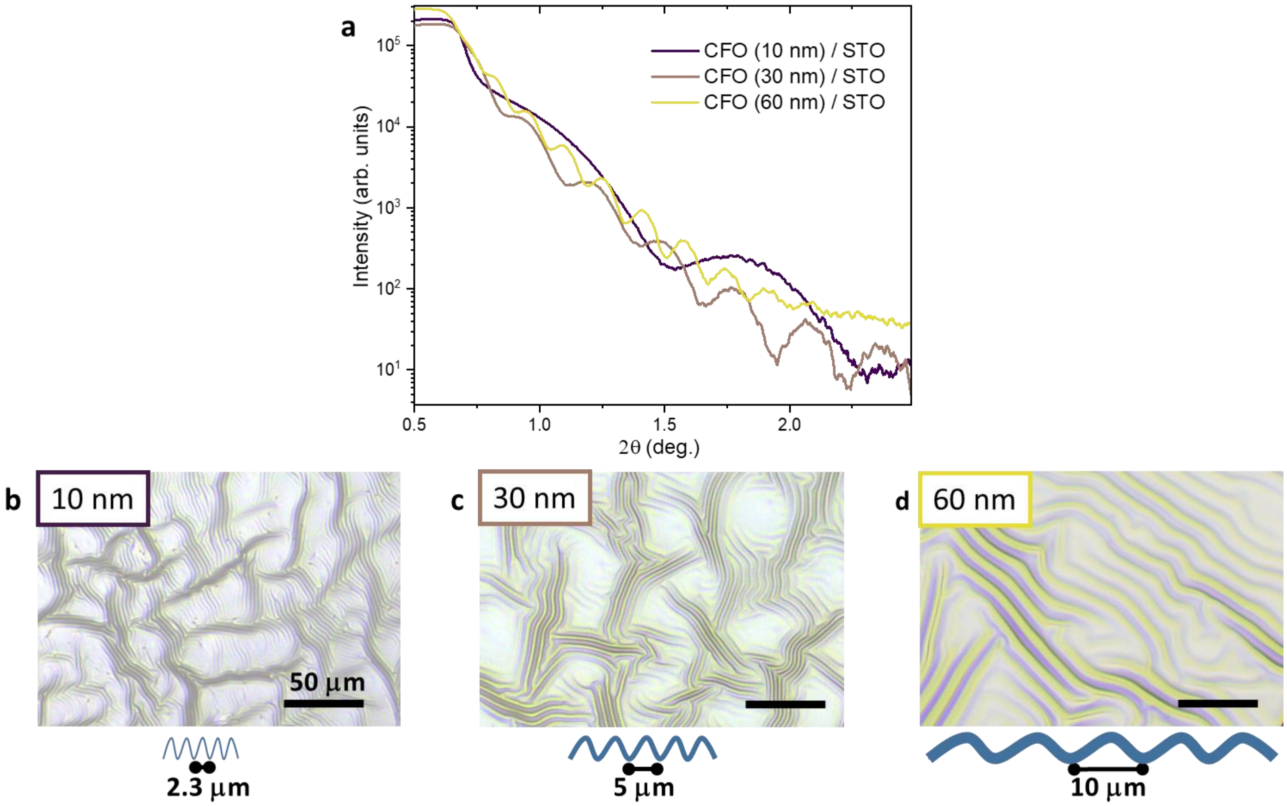

Figure S9: CFO membranes of different thicknesses. (a) XRR measurements for thickness determination of CFO film grown on STO. (b-d) Optical microscopy images of rippled CFO membranes of different thickness on PET polymer, scale bar of 50 μm. Schematic proportional representation of the ripples are made for comparison.

References

(1) Salles, P.; Caño, I.; Guzman, R.; Dore, C.; Mihi, A.; Zhou, W.; Coll, M. Facile Chemical Route to Prepare Water Soluble Epitaxial $Sr_3Al_2O_6$ Sacrificial Layers for Free-Standing Oxides. *Adv. Mater. Interfaces*, **2021**, *8*, 2001643.

(2) Koster, G.; Kropman, B. L.; Rijnders, G. J. H. M.; Blank, D. H. A.; Rogalla, H. Quasi-ideal Strontium Titanate Crystal Surfaces Through Formation of Strontium Hydroxide. *Appl. Phys. Lett.*, **1998**, *73*, 2920–2922.

(3) Chen, X.-D.; Liu, Z.-B.; Zheng, C.-Y.; Xing, F.; Yan, X.-Q.; Chen, Y.; Tian, J.-G. High-quality and Efficient Transfer of Large-Area Graphene Films Onto Different Substrates.

Carbon **2013**, *56*, 271–278.

(4) Cendula, P.; Kiravittaya, S.; Mei, Y. F.; Deneke, C.; Schmidt, O. G. Bending and Wrinkling as Competing Relaxation Pathways for Strained Free-Hanging Films. *Phys. Rev. B* **2009**, *79*, 085429.

(5) Melzer, M.; Lin, G.; Makarov, D.; Schmidt, O. G. Stretchable Spin Valves on Elastomer Membranes by Predetermined Periodic Fracture and Random Wrinkling. *Adv. Mater.*, **2012**, *24*, 6468–6472.
